# P-2040. Real-world safety and effectiveness of ensitrelvir in COVID-19 patients with risk factor : a post-marketing surveillance in Japan

**DOI:** 10.1093/ofid/ofae631.2196

**Published:** 2025-01-29

**Authors:** Satoru Takashima, Noriko Hayashi, Eri Tsukimura, Eriko Ogura

**Affiliations:** Shionogi & Co., Ltd., Osaka, Osaka, Japan; Shionogi & Co., Ltd., Osaka, Osaka, Japan; Shionogi Pharmacovigilance Center Co., Ltd., Osaka, Osaka, Japan; Shionogi & Co., Ltd., Osaka, Osaka, Japan

## Abstract

**Background:**

After obtaining emergency approval on November 22, 2022, a post-marketing surveillance (PMS) in COVID-19 patients was conducted in Japan to investigate the safety and effectiveness of ensitrelvir. We report the safety and effectiveness of ensitrelvir focusing on patients with high-risk (HR) factors for severe COVID-19.

**Methods:**

In the PMS, COVID-19 patients administered ensitrelvir for the first time in about 500 Japanese hospitals or clinics were targeted and observed for 28 days after the first administration. Patient demographics, concomitant medications, other therapy for COVID-19, adverse events, and clinical course (fever, systemic, respiratory, and gastrointestinal symptoms) were investigated.

**Results:**

A total of 4155 cases participated from November 2022 to August 2023, and 4125 case reports were collected. The safety and effectiveness analysis population were 3760 and 3638 cases respectively. Among the safety analysis population, the average age was 43.6 years; 452 cases (12.0%) were aged 65 and over; 1823 cases (48.5%) were male; and 939 cases (25.0%) had HR factors. The severity of COVID-19 was mild in 3666 cases (97.5%), moderate 1 in 81 cases (2.2%), moderate 2 in 3 cases (0.1%), and asymptomatic in 10 cases (0.3%).

In the safety analysis population, 379 events of non-serious adverse drug reactions (ADRs) were reported, and 5 events (headache, nausea, vomiting, cold sweat, and generalized oedema) of serious ADRs were reported. The major ADRs in the safety analysis population were diarrhoea in 91 cases (2.4%), nausea in 43 cases (1.1%), headache in 42 cases (1.1%), vomiting in 24 cases (0.6%), and rash in 20 cases (0.5%). The frequency of ADRs was 7.09% in the standard-risk (SR) group and 7.56% in the HR group. The median time to resolution of fever was 48.0 hours in the SR group (n=1929) and 36.0 hours in the HR group (n=590). The median time to resolution of all symptoms was 204.0 hours in the SR group (n=2732) and 156.0 hours in the HR group (n=903). Thirteen cases (0.4%) were hospitalized, and the reason was exacerbation of COVID-19 in 10 cases, and others in 5 cases.

**Conclusion:**

Results of the analysis suggest that ensitrelvir is well tolerated and effective in patients with risk factors same with patients without risk factors.

**Disclosures:**

Satoru Takashima, n/a, Shionogi & Co., Ltd: Stocks/Bonds (Private Company) Noriko Hayashi, n/a, Shionogi & Co., Ltd: Stocks/Bonds (Private Company) Eri Tsukimura, n/a, Shionogi & Co., Ltd: Stocks/Bonds (Private Company) Eriko Ogura, MD, Shionogi & Co., Ltd: Employee

